# Acute stress-induced change in polysialic acid levels mediated by sialidase in mouse brain

**DOI:** 10.1038/s41598-019-46240-6

**Published:** 2019-07-09

**Authors:** Chikara Abe, Yang Yi, Masaya Hane, Ken Kitajima, Chihiro Sato

**Affiliations:** Bioscience and Biotechnology Center, Nagoya University, Chikusa, Nagoya 464-8601 Japan

**Keywords:** Stress and resilience, Glycobiology

## Abstract

Stress is an important environmental factor influencing human behaviour and causing several mental disorders. Alterations in the structure of polysialic acid (polySia/PSA) due to genetic alterations in ST8SIA2, which encodes a polySia-synthesizing enzyme, are related to certain mental disorders. However, whether stress as an environmental factor leads to changes in polySia structure is unknown. Here we studied the effects of acute stress on polySia expression and found reductions in both the quantity and quality of polySia in the olfactory bulb and prefrontal cortex, even with short-term exposure to acute stress. The use of inhibitors for sialidase, microglia and astrocytes revealed that these declines were due to a transient action of sialidase from microglia and astrocytes in the olfactory bulb and prefrontal cortex, respectively. These data suggest that sialidase dynamically regulates polySia expression in a brain region-specific manner.

## Introduction

Mental disorders including schizophrenia (SCZ), bipolar disorder (BD), autism spectrum disorder (ASD), major depression disorder, attention deficit hyperactivity disorder, anxiety disorders and drug and alcohol abuse are becoming global problems^1^. A significant percentage of the human population suffers from these disorders and socio-economic problems associated with these disorders are increasing worldwide. Improvements in the instruments used for sequencing and bioinformatic analyses have revealed many candidate genes associated with the development of these disorders. These include dysbindin (DTNBP1)^2^, which is a part of the protein complex associated with lysosome-related organelles complex 1; AKT serine/threonine kinase 1 (AKT-1)^3^; catechol-*O*-methyltransferase (COMT)^4,5^, and dopamine receptor D2 (DRD2)^5^; disrupted in schizophrenia 1 (DISC1)^6^, which was first identified in Scottish families and is thought to encode a scaffold protein for nuclear distribution element-like 1 (NDEL1)^7^ and 14-3-3(YWHAD)^8^; neuregulin 1 (NRG1)^9^ and a deletion of chromosome 22q11^10^. Researches during the five decades have clearly demonstrated that no single gene is causative by itself and that there are multiple susceptibility genes, single nucleotide polymorphisms (SNPs) and rare chromosomal rearrangements involving deletions, duplications, inversions or translocations of deoxyribonucleic acid (DNA), each of which contributes to an incremental risk for the development of mental disorders^10,11^. In addition to genetic risk factors, environmental risk factors are important susceptibility factors. Multiple, and possibly even individually-specific personalized, factors increase the risk of development of mental disorders^10^. Therefore, it is important to understand the disorders from both the genetic and environmental perspectives.

One candidate molecule in mental disorders is polysialic acid (polySia/PSA). PolySia is a brain-specific unique glycan that is highly regulated developmentally in mammalian brains. Expression of polysialylated neural cell adhesion molecule (NCAM) (polySia-NCAM) in mice has been well studied^12,13^. In mice, polySia expression begins at embryonic day 9.5, increases until just before birth and dramatically decreases between 10 days to 3 weeks after birth. At 8 weeks, polySia almost disappears, but remains in restricted areas where neurogenesis is ongoing, such as the olfactory bulb (OB) and hippocampus (HIP). Therefore, polySia is considered to be a marker for neurogenesis in these regions^12,13^. In addition to the OB and HIP, intense staining of polySia-NCAM in adult brain is also observed in the amygdala (AMG), suprachiasmatic nucleus (SCN) and prefrontal cortex (PFC)^14^. The molecular mechanism and biological meaning of polySia expression at restricted area even in adult brain is unknown.

The first report that clearly demonstrated the relationship between polySia-expressing cells and SCZ was published in 1995. The authors described that the number of polySia- immunostained cells derived from the hilus region of the HIP in the brains of SCZ patients was lower as compared to that in normal brains^15^. Later, reduced polySia-NCAM expression was detected in layers IV and V of the dorsolateral PFC in SCZ patients^16^. No such difference was observed in the AMG^17^, implicating region-specific polySia impairment (reduction of polySia-immunostained cells) as a feature of SCZ. In the AMG of BD patients, upregulated expression of polySia was observed. PolySia-NCAM was significantly decreased in the lateral amygdala and in the basolateral and basomedial amygdala of patients with depression. These data indicate that change in polySia expression in disorder-specific regions of the brain may be a feature of mental disorders, although the mechanism is unknown. The presence of a 70 kDa NCAM fragment in serum was positively correlated with the severity of negative symptoms in SCZ (type II)^18^. More recently, the level of polySia-NCAM in serum was found to be increased in SCZ; in patients with negative symptoms, serum polySia-NCAM was associated with a decreased volume of Brodmann area 46 in the left PFC, with an unknown origin of this protein^19^. Therefore, impairment of polySia-NCAM expression is a feature of the brain of patients suffering from mental disorders.

The polysialyltransferase, ST8 alpha-*N*-acetyl-neuraminide alpha-2,8-sialyltransferase 2 (ST8SIA2) is responsible for polySia synthesis^20^. A relationship between mental disorders and SNPs of *ST8SIA2* has been demonstrated^21^. In addition, biochemical analyses revealed the involvement of some of these SNPs in the structural and functional impairment of polySia^21,22^. For example, the rs545681995 SNP leads to a single amino acid change and the resulting mutated ST8SIA2 led to reduced polySia synthetic activity and impaired quantity and length of polySia^23,24^. In addition, the binding affinity of polySia to brain-derived neurotrophic factor (BDNF), fibroblast growth factor 2 (FGF2) and dopamine (DA) was drastically impaired^24,25^. In contrast, the rs2168351 SNP present in BD patients was associated with the upregulated expression of polySia^26^. All these data are consistent with the reported reduction of the level of polySia-NCAM in the brains of SCZ patients^15–17^. *St8sia2*-knockout (KO) mice are shown to be a suitable model for SCZ. These mice display impaired working memory, deficits in prepulse inhibition, anhedonic behaviour and increased sensitivity to amphetamine-induced hyperlocomotion^27^.

In contrast to the plethora of studies concerning genetic factors, the influence of environmental factors on polySia expression in the aetiology of mental disorders like SCZ and depression is less reported. Studies are ongoing, prompted by the view that polySia is an important marker for neurogenesis. One of these environmental factors is stress. Stress is considered a primary risk factor for most mental disorders^28^ and the effects of chronic stress on polySia expression, especially in the HIP, PFC and AMG, have been demonstrated^29–31^. However, the effects of acute stress as an environmental factor on polySia expression has not been studied at all, even though acute stress is an important environmental factor that can initiate chronic stress and is involved in mental disorders^32^ that include SCZ, BD, depression, and post-traumatic stress disorder.

To explore the effects of acute stress on polySia expression in specific regions of the brain, mice were suspended by their tails to induce acute stress, followed by the analysis of polySia-NCAM expression in brain tissues. The findings revealed a new mechanism of acute stress-induced decrease in polySia expression involving sialidase in the OB and PFC.

## Results

### Effect of acute stress on polySia expression in mouse brain

**Figure 1:**
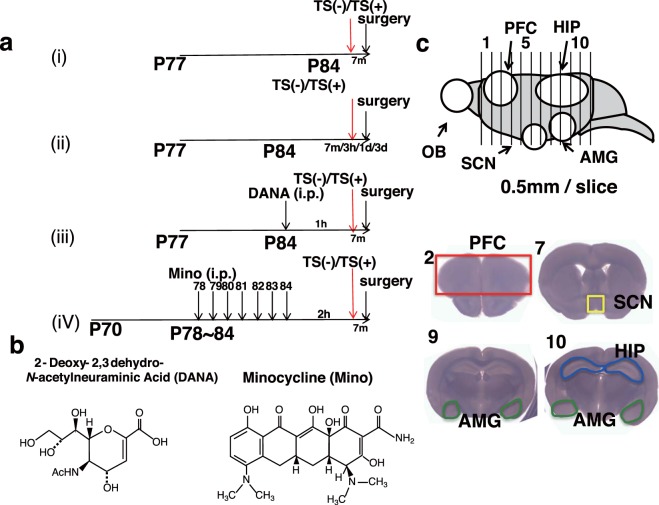
Experimental schedules. (**a**) Time schedule of mice experiments. All mice (10 weeks, P77 or P70) used for the experiments were housed over 1 week (P84). Tail suspension (TS) as acute stress for 7 min was performed. Immediately after TS, brains were excised (i). (ii) Time course effect of TS on polySia expression. Mice were sacrificed at 7 min, 3 h, 1 day or 3 days after TS. (iii) Sialidase inhibition analysis. TS was performed on mice at 1 h after saline or sialidase inhibitor (2-deoxy-2,3-dehydro-*N*-acetylneuraminic acid (DANA) injection. (iv) Microglia inhibition analysis. TS was performed on mice after saline or minocycline (Mino) injection. For inhibition of microglia, Mino injection was given once per day for 7 days and TS was performed 2 hours after final injection. As a control, saline was injected. (**b**) Chemical structure of DANA and Mino. (**c**) Strategy of brain sections. Brains were sliced into 0.5 mm/slice except olfactory bulb and 12 sections were prepared (*Upper panel*). Brain areas in sections used for this experiment are shown and include PFC from section 2, SCN from section 7. AMG from section 9 and 10. HIP from section 10. These areas were separated and used for the experiment.

**Figure 2:**
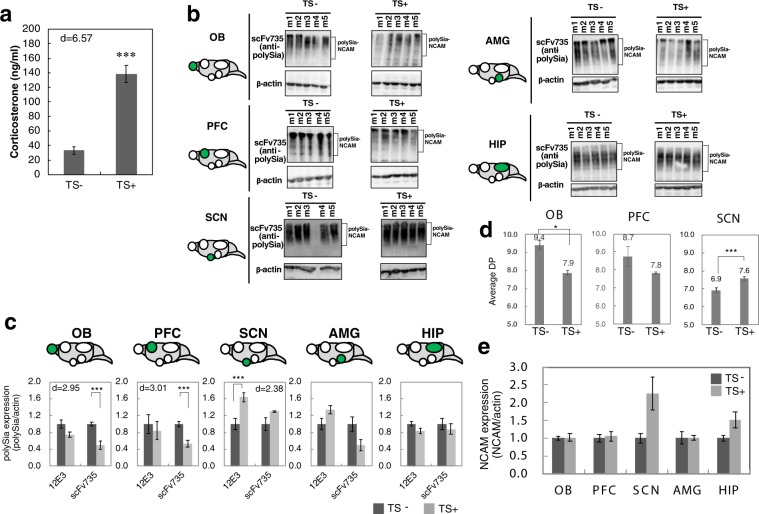
Effect of TS as an acute stress. (**a**) Concentrations of corticosterone in serum before and after TS (n = 3 mice; ***p < 0.005; d = 6.57) (**b**) PolySia expression in the OB, PFC, SCN, AMG, and HIP before and after TS as the acute stress. Homogenates derived from the five brain areas were analyzed using anti-polySia antibody (scFv735) and anti-β-actin antibody (n = 5 mice). For each brain area, the blots for TS− and TS+ were cut out from a larger blot obtained by the same SDS-PAGE/immunoblotting. The each blot was further cut into the upper and lower parts for anti-polySia and anti-actin antibodies, respectively. The original whole blots corresponding to the blots for each brain area are shown in Supplementary Fig. S1b. The square brackets indicate polySia-NCAM. (**c**) Quantification of polySia. The polySia expression was evaluated by the intensities of anti-polySia antibody and anti-β-actin antibody as shown in (**b**). Each of the western blottings was repeated 3 times and the error bars show the standard error (SE). TS− was set to 1.0. (n = 5 mice, t-test, ***p < 0.005). (**d**) Chemical analyses of the change of polySia structure. The amount of α2,8-linked polySia structure was evaluated by fluorometric C_7_/C_9_ analyses and the average DP is shown (n = 3 mice, t-test, *p < 0.05, ***p < 0.005). (**e**) NCAM expression in OB, PFC, SCN, AMG, and HIP before and after TS as acute stress. Homogenates derived from 5 brain areas after Endo-N were analyzed using anti-NCAM antibody and anti-β-actin antibody (n = 5 mice for OB, PFC, AMG, n = 4 mice for HIP, n = 3 mice for SCN). Each of the western blottings was repeated 3 times and the error bars show the SE. The original blots were shown in Supplementary Fig. S1c.

To understand the influence of acute stress on polySia expression in several regions of the brain, the tail suspension (TS) test (TST)^33^ was performed (Fig. 1a(i)). The strain value of 60% ± 10 was consistent with previously reported results^34^. To confirm that tail suspension did impose stress, the serum concentrations of corticosterone were determined before and after the test. The approximately 3-fold increase in the level of corticosterone after the test (Fig. 2a) confirmed the induction of acute stress in the mice.

Next, we evaluated the amount of polySia in five regions of the brain (OB, PFC, SCN, AMG and HIP) by western blotting and immunostaining using anti-polySia antibodies (Fig. 2b and Supplemental Fig. S1). Relatively more polySia was expressed in adult mice, even though OB and HIP are well-known polySia-expressing regions. Interestingly, altered polySia expression was observed in the OB, PFC and SCN after exposure to acute stress significantly. In the OB and PFC, polySia was decreased, as detected using the single chain variable fragment (scFv)735 antibody (which recognizes polySia larger than 11-mer^35^). AMG has the same tendency. By contrast, polySia was increased in the SCN, as detected using the 12E3 antibody that recognizes polySia larger than 6-mer including the non-reducing terminal end^35^ (Fig. 2c). Tissue staining corroborated the results (Supplemental Fig. S1). In addition, the average degree of polymerization (DP) of polySia evaluated by fluorometric 7-carbon sugar (C_7_)/9-carbon sugar (C_9_) analysis^36^, an established chemical detection method for α2, 8-linked Neu5Ac, revealed decreases in the OB and PFC, and increase in SCN after acute stress (Fig. 2d). Next, we analysed the NCAM amounts (Supplemental Fig. S1) and found that no significant changes of NCAM was observed in PFC and OB (Fig. 2e). The collective data suggested that acute stress could change polySia expression specifically in the brain.

**Figure 3:**
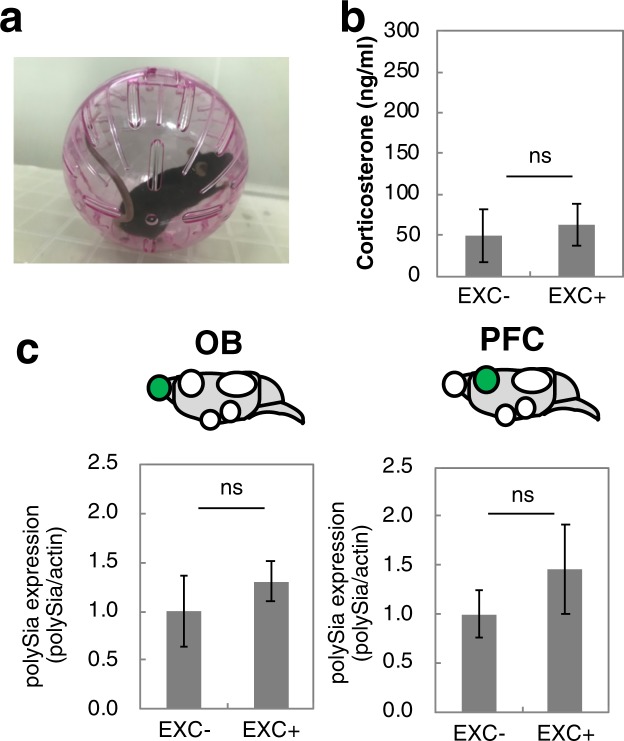
Effect of exercise on polySia expression in mice brains. (**a**) Photo of rolling bowl used in the exercise experiment. (**b**) Concentrations of corticosterone in serum before and after the 3-min exercise (EXC) (n = 3 mice). (**c**) Quantification of polySia. PolySia expression was evaluated by the intensities of anti-polySia antibody and anti-β-actin antibody as shown in Supplementary Fig. S2. Each of the western blottings was repeated 3 times and the error bars show the SE. TS− was set to 1.0. (n = 3 mice). ns indicates no significance.

**Figure 4:**
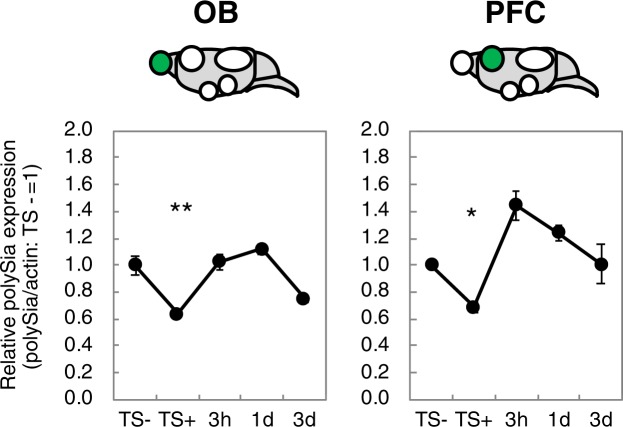
Time course of acute stress-induced polySia decrease. The time course of acute stress-induced polySia decrease was evaluated by the staining with anti-polySia antibody and anti-β-actin antibody. Each of the western blottings was repeated 3 times and the error bars show the SE. TS− indicates polySia expression before TS. TS+, 3 h, 1 day, and 3 days indicate the polySia expression 7 min, 3 h, 1 day and 3 days after TS treatment, respectively. TS− was set to 1.0. (n = 4 mice, *p < 0.05, **p < 0.01 vs TS−).

It is reported that the transient upregulation of corticosterone concentration does not influence polySia expression in the OB, PFC, SCN, AMG and HIP regions of the brain in the short-term^37^. However, whether vigorous movement, such as occurs during the TS, influences polySia expression is unclear. To assess the influence of movement on the expression of polySia, mice were exercised in a rolling bowl in the absence of stress for 3 min (Fig. 3a), which is the average time of struggle during the TS. The absence of stress was confirmed by determination of corticosterone concentration (Fig. 3b). In this experiment, polySia expression was similar before and after exercise (EXC) (Fig. 3c and Supplementary Fig. S2). The findings indicated that decrease polySia expression was not related to exercise or corticosterone level, but rather to the acute stress. To understand the time course of polySia expression after acute stress, the TS was performed and OB and PFC tissues were collected immediately afterward or 3 h, 1 day and 3 days later for the determination of polySia expression (Fig. 1a(ii)). The decrease in polySia was recovered within 3 h in the OB and within 1 to 3 days after acute stress in the PFC (Fig. 4). These results supported the view that polySia expression is dynamic and highly regulated in the brain.

**Figure 5:**
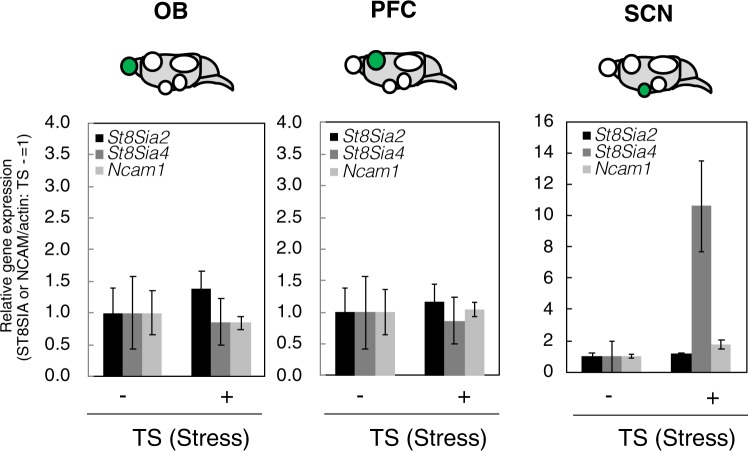
Real-time PCR of polySia-related genes before and after acute stress. The amounts of polySia-related genes, *St8sia2*, *St8sia4*, and *Ncam1* in OB, PFC and SCN were evaluated by real-time PCR. Gene expression of TS− (without acute stress) was set to 1.0. (n = 3 mice, t-test).

To understand the mechanism of change in polySia induced by acute stress, we analysed the expression levels of the polySia-related genes *St8sia2/sialyltransferase-X (STX)*, *ST8 alpha-N-acetyl-neuraminide alpha-2*,*8-sialyltransferase 4 (St8sia4)/polysialyltransferase-1(PST)* and *NCAM* by real-time polymerase chain reaction (PCR). *St8sia2/STX* and *St8sia4/PST* encode two polysialyltransferases that are responsible for the synthesis of polySia^38^. *NCAM* encodes NCAM, which is the major carrier protein of polySia in brain (approximately 90%). Interestingly, *St8sia2/STX*, *St8sia4/PST* and *NCAM* expressions were unchanged in the PFC and OB following acute stress (Fig. 5). Thus, the observed decrease in polySia expression during the period of acute stress in the TS was not due to the change of polySia-related gene expression, but rather to some other mechanism. By contrast, altered expression of *St8Sia4/PST* induced by acute stress was evident in the SCN, indicating that the upregulated expression of polySia observed in the SCN after the acute stress of the TS was due to the upregulated expression of *St8sia4/PST*.

### Effect of 2-deoxy-2,3-dehydro-*N*-acetyl-neuraminic acid on acute stress-induced polySia expression

We next explored the possible involvement of sialidase and/or microglia in the decreased polySia in the OB and PFC induced by acute stress, based on the observation that polySia is degraded by sialidase in exosomes secreted from the microglia^39^. This was confirmed (Fig. 1b) using 2-deoxy-2,3-dehydro-*N*-acetyl-neuraminic acid (Neu5Ac2en/DANA), which inhibits sialidase (neuraminidase) activity by competitive inhibition^40^ toward all types of neuraminidase (Neu) 1-Neu4^41^, and using minocycline, which inhibits microglia activation^42^ by blocking the translocation of nuclear factor-kappa B^43^.

**Figure 6:**
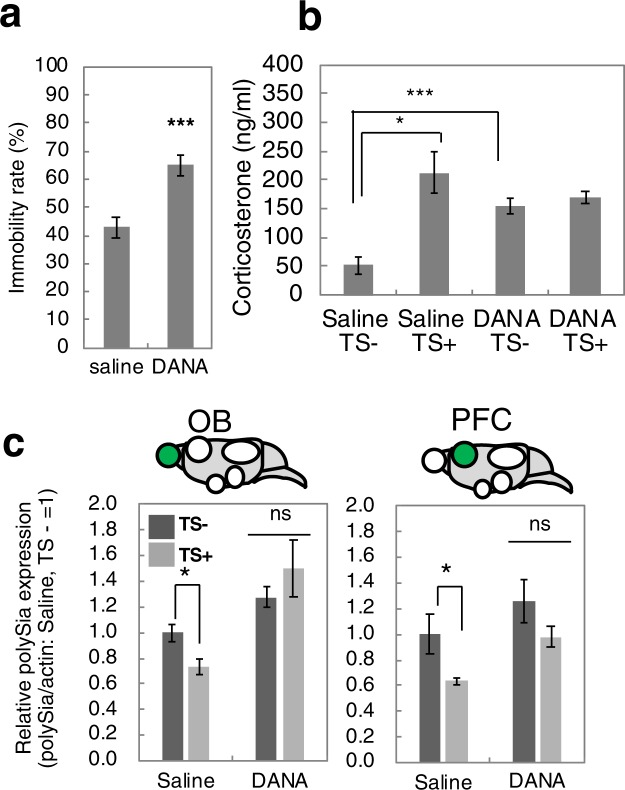
Effects of sialidase inhibitor on polySia expression during acute stress. (**a**) Immobility rate of the TST. Mice injected with saline or sialidase inhibitor DANA were exposed to the TST and the immobility rate was measured. (n = 4 mice, t-test, *p < 0.05) (**b**) Concentrations of corticosterone in serum. Mice were pretreated with saline or DANA. Then half of the mice were exposed to acute stress (TS+) and the other half were not exposed (TS−). (n = 5 mice, *p < 0.05, ***p < 0.005) (**c**) Quantification of polySia. PolySia expression in OB and PFC was evaluated by western blotting as shown in Supplementary Fig. S4b. Each of the western blottings was repeated 3 times and the error bars show the SE. Saline/TS− was set to 1.0 (n = 5 mice, t-test, *p < 0.05).

Mice were divided into four groups (n = 5 per group): injection with DANA followed by acute stress (Fig. 1a(iii)), injection of saline followed by acute stress, injection with DANA and no acute stress, and injection of saline and no acute stress. The influence of DANA on the depression state was assessed by measuring the immobility time during tail suspension (Fig. 6a). DANA upregulated the immobility rate after tail suspension as compared with saline injection (Fig. 6a). The subsequent determination of the concentration of corticosterone revealed that DANA injection upregulated the secretion of corticosterone (Fig. 6b). This finding is the first demonstration that the sialidase inhibitor DANA can regulate corticosterone concentration in the serum. The mechanism of the regulation is unknown. Analysis of the change in sialidase level due to DANA injection revealed down-regulation of sialidase activity in the PFC and OB (Supplementary Fig. S3a). Analysis of polySia expression revealed that the acute stress-induced decrease in polySia expression observed in the PFC and OB in mice injected with saline (Fig. 6c and Supplementary Fig. S3b) did not change significantly after DANA injection (Fig. 6d), clearly indicating the involvement of sialidase in this acute stress-induced phenomenon. We further analysed the expression of sialidase genes assessed by real-time PCR and it was not significantly changed (Supplementary Fig. S4).

### Effect of minocycline on polySia expression induced by acute stress

**Figure 7:**
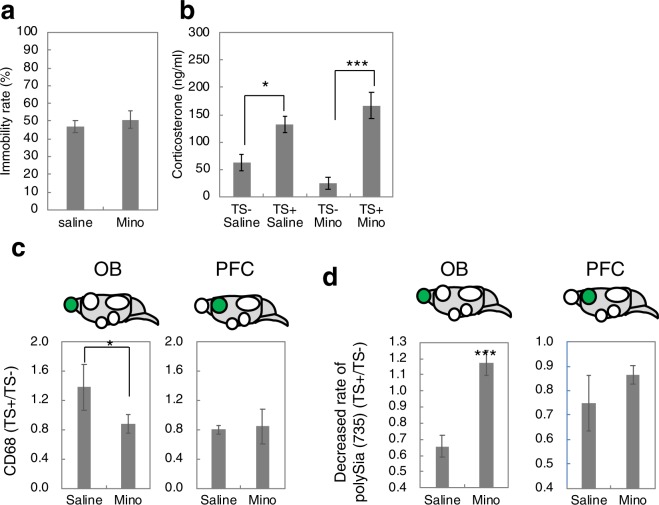
Effects of microglia inhibitor injection on polySia expression during acute stress. (**a**) Immobility rate of TST. Mice injected with saline or microglia inhibitor, Mino were used for the TST and the immobility rate was measured (n = 5 mice). (**b**) Concentrations of corticosterone in serum. Mice were pretreated with saline or Mino. Half of the mice were exposed to acute stress (TS+) and the other half were not exposed (TS−) (n = 5 mice, *p < 0.05, ***p < 0.005). (**c**) Relative ratio of activated microglia as CD68 staining. CD68 staining in the OB and PFC derived from saline treated (Saline) and minocycline-treated mice (Mino) was evaluated before and after acute stress (TS− and TS+) as shown in Supplementary Fig. S5b and relative ratio of activated microglia (TS+/TS−) are shown. (**d**)The ratio of acute stress-induced polySia decrease. PolySia expression in OB and PFC were evaluated by western blotting as shown in Supplementary Fig. S5c. Each of the western blottings was repeated 3 times and the error bars show the SE.

**Figure 8:**
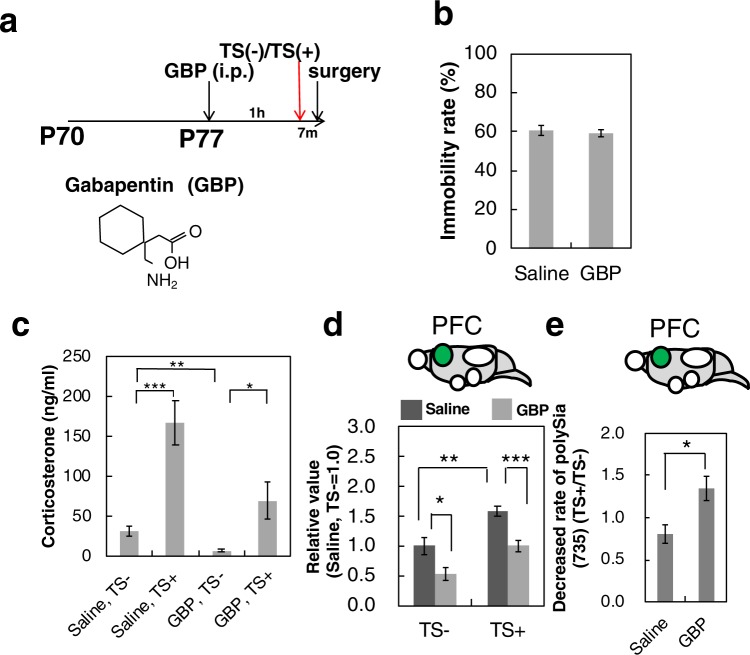
Effects of astrocyte inhibitor injection on polySia expression during acute stress. (**a**) Time schedule of mice experiments. Mice (10 weeks, P77) used for the experiments were housed over 1 week (P84). TS as acute stress for 7 min was performed and immediately after TS, brains were excised. TS was performed on mice at 1 h after saline or GBP injection. Lower panel shows the chemical structure of GBP. (**b**) Immobility rate. Mice injected with saline or GBP were exposed to TST and the immobility rate was measured (n = 5 mice). (**c**) Concentrations of corticosterone in serum. Mice were pretreated with saline or GBP. Half of the mice were exposed to acute stress (TS+) and the other half were not exposed (TS−) (n = 5 mice, *p < 0.05, ***p < 0.005). (**d**) Relative ratio of GFAP staining derived from astrocyte as GFAP staining. GFAP staining in PFC derived from saline-treated (Saline) and GBP-treated mice was evaluated before and after acute stress (TS− and TS+) as shown in Fig. S6a and relative ratio of GFAP (TS+/TS−) has been shown. (**e**) The ratio of acute stress-induced polySia decrease. PolySia expression in PFC were evaluated by western blotting as shown in Fig. S6b. Each of the western blottings was repeated 3 times and the error bars show the SE.

To assess if microglia activation was also involved the acute stress-induced decease of polySia, minocycline was used to inactivate microglia. Mice were divided into four groups (n = 5 per group): injection of minocycline followed 7 days later by acute stress, injection of saline followed 7 days later by acute stress, injection of minocycline alone and injection of saline alone (Fig. 1a(iv)). The immobilization rates of mice injected with saline or minocycline were the same (Fig. 7a). The concentration of corticosterone in minocycline-injected mice was similarly upregulated as in saline-injected mice (Fig. 7b). The concentration of corticosterone before acute stress exposure was decreased in mice prior to minocycline injection compared to the concentration in mice prior to saline injection, indicating the involvement of the microglia in the secretion of corticosterone in normal conditions. Western blot results revealed the inactivation of the microglia (Fig. 7c and Supplementary Fig. S5a), although this was significant only in the OB. Next, we analysed polySia expression (Fig. 7d and Supplementary Fig. S5b) and found that the decrease in polySia expression induced by acute stress was impaired in the OB, but not the PFC, in minocycline-injected mice. To understand the sialidase-induced polySia decrease in the PFC, we further analysed the possibility of the involvement of astrocytes using gabapentin (GBP) (Fig. 8a) as an astrocyte inhibitor^44^. The immobility rate and corticosterone concentration were normal following gabapentin injection and prior to acute stress (Fig. 8b,c). The injection of gabapentin decreased the expression of glial fibrillary acidic protein (GFAP) (Fig. 8d and Supplementary Fig. S6a). Analysis of polySia expression in the PFC (Fig. 8e and Supplementary Fig. S6b) revealed the absence of the acute stress-induced polySia decrease following gabapentin injection, compared to the decrease observed following saline injection (Fig. 8e, saline). The collective data demonstrated that the polySia decrease induced by acute stress was due to the astrocyte-related effect of sialidase.

## Discussion

**Figure 9:**
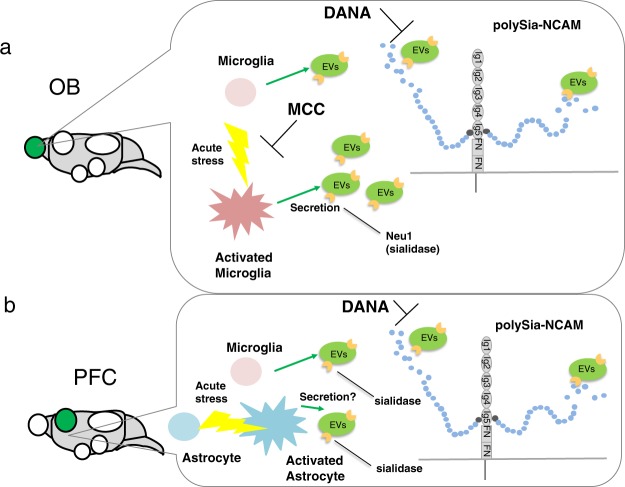
Hypothesized mechanism of acute stress-induced polySia decrease. (**a**) In the OB, microglia are activated by acute stress. These activated microglia released sialidase probably from exosomes^39^ and sialidase cleaves polySia immediately based on the observation that DANA and Mino suppressed the acute stress-induced polySia decrease. (**b**) In PFC, polySia was cleaved, however, the cleavage was only inhibited by sialidase inhibitor, but not microglia inhibitor, indicating that other cells like activated astrocyte may be involved in this phenomenon.

In this study, we quantitatively and qualitatively evaluated the change in polySia expression at specific regions of the murine brain after very short exposure to acute stress. Especially, a significant decrease in polySia expression in the OB and PFC was observed using the scFv735 polySia antibody directed at the long-chain polySia structure^35^. This was also confirmed by a chemical analysis^36^. In the SCN, increased polySia expression was revealed using 12E3 polySia antibody directed at the polySia chains having more than six units including the non-reducing terminal end^35^. These data collectively demonstrate that polySia expression in the brain is not static, but instead is dynamic in region- and condition-specific manners. A hint of the underlying mechanism of this rapid decrease in polySia expression in mouse brain came from the observation of the rapid clearance of polySia in microglia cells after lipopolysaccharide stimulation^39^. We reported that polySia chain in neural cells rapidly decreased when co-incubated with microglia cells after lipopolysaccharide treatment; it appeared that the rapid clearance of polySia was due to the sialidase Neu1 on the exovesicles secreted from activated microglia. Based on the observation of the rapid clearance of polySia in microglia cells, we hypothesized that such clearance might occur in the brain after stimulation by stress because microglia are easily activated in stressful conditions^45^. The present data confirmed that the rapid decrease in polySia expression after acute stress is due to the sialidase in the OB and PFC, because inhibition of sialidase by DANA clearly inhibited the acute stress-induced decrease in polySia expression (Fig. 6c). Especially, in the OB, minocycline-mediated inhibition of microglia activation (Fig. 7d) confirmed that sialidase is derived from activated microglia after exposure to acute stress (Fig. 9). In the PFC, the decrease was inhibited by DANA, but not by minocycline. It was inhibited by gabapentin, an inhibitor of astrocyte activation^44,46^ (Fig. 8), indicating that sialidase originated from astrocytes in the PFC (Fig. 9). This should be further confirmed by cell-based assays with astrocyte like microglial cell lines.

Another interesting observation was the recovery of the acute stress-induced decrease in polySia expression between 3 and 24 h. This recovery indicated that polySia expression is highly regulated homeostatically. In a previous study, sialidase activity derived from microglia cells transiently appeared and disappeared within 30 min^39^. This might be the reason for the transient decrease in polySia expression. The finding of a dynamic change in the polySia structure under acute stress conditions highlights the need to pay attention to the timing of sampling when polySia staining techniques are employed in brain research. The 7 min exposure to acute stress clearly changed the expression of polySia; this may explain the contradictory polySia staining sometimes observed in the human brain. In mice, the results are usually consistent because the conditions under which the mice are maintained can be regulated. This sort of regulation is difficult to achieve in case of humans. Therefore, it is necessary to consider the possibility of dynamic change in polySia staining due to variable conditions, because polySia expression is very sensitive, especially in the OB, PFC and SCN after exposure to stress, even for only several minutes. The OB and HIP are recognized regions where adult neurogenesis occurs and polySia is sometimes used to monitor adult neurogenesis^12^, although the biological significance of the presence of polySia remains unclear and cells that express polySia are really newly-synthesized cells. Presently, the distribution of polySia was significantly large in the five regions examined. Some of these areas are devoid of newly synthesizing cells. Further examination of polySia-expressing cells in each region will be instructive. Another important note concerning polySia structure in the brain research is that the different specificity of the 735 and 12E3 polySia antibodies in terms of DP and the non-reducing terminal Sia on polySia^35^. The 735 antibody can recognize longer polySia chains than 12E3. By contrast, 12E3 binds to the non-reducing terminal Sia in the polySia chain, and so is unable to detect the change in the chain longer length of polySia (i.e., *de novo* synthesis and degradation). Thus, to precisely elucidate the changing state of polySia it is necessary to understand the specificities of the polySia antibodies. As shown in Fig. 2, the change in polySia staining after acute stress was observed using 735, but not 12E3. These results indicate that the cleavage of polySia happens only in longer polySia chains in the OB and PFC. PolySia can bind to neurologically active molecules such as BDNF, FGF2 and dopamine^47^. In addition, sialidase action resulted in secretion of pre-retained BDNF from the polySia chains^39^ because longer polySia chains, not shorter oligoSia chains, bind only to BDNF^48^. These data suggest that the transient increase of sialidase levels leads to the secretion of BDNF and other neurologically important molecules to handle stress conditions by changing longer polySia to shorter polySia chains. Therefore, these phenomena might be important to cope with stress conditions. On the other hand, an increase in polySia staining using the 12E3 antibody was observed in SCN as was the upregulated expression of *St8sia4*. The increased polySia expression on the cell surface of SCN may contribute to the homeostatic functions in SCN that *St8sia4* might be involved with.

The relationship between polySia and/or the *ST8SIA2* and mental disorders was recently reported^21^. In the brains of patients with SCZ, polySia expression is decreased in the PFC^16^ and HIP^15^. Interestingly, several significant SNPs of the *ST8SIA2* that express an enzyme responsible for polySia synthesis are reportedly related to SCZ and BD^21^. Precise biochemical examination of the significant SNPs revealed changes in polySia expression, structure and functions^21^. In addition, *St8sia2*-KO mice have been reported to be a model of SCZ^27^. These data implicate polySia as a molecule that could be useful to monitor these diseases from a genetic viewpoint. The effects of environmental factors like stress on polySia expression were also analysed. Both acute and chronic stress change polySia expression at particular regions of the mouse brain. In case of chronic stress, several analyses revealed the decreased polySia expression, especially in HIP; this decrease can impair adult neurogenesis^49,50^. PolySia is a marker of neurogenesis and prolonged exposure to corticosterone derived from the hypothalamus-pituitary-adrenal (HPA) axis influences neurogenesis and destroys tissues. We previously demonstrated that transient increase of corticosterone levels did not influence the quantity of polySia detected by the 12E3 antibody in any region of the brain mice^37^. Therefore, it is considered that prolonged exposure to corticosterone is a key requirement for the impairment of polySia-NCAM and HIP tissues. We found, however, that the transiently increased corticosterone concentration after acute stress decreased within 2 hours, indicating that repeated exposure of corticosterone might be the cause of the decreased expression of polySia-NCAM in HIP, as reported previously. In this study, we focused on the effects of acute stress (7 min) on polySia expression. This has not been studied before. Surprisingly, we found that polySia expression changed only after the 7-min acute stress, which is the shortest time course of polySia expression analysed so far. The transient increase of corticosterone level^37^ and exercise (Fig. 3) did not decrease polySia expression in the OB and PFC, indicating that the change of polySia expression was due to an unknown cause. One cause might be microglia activation by stress, probably via the sensory nervous system. Microglia are easily activated by corticosterone^51^. However, the present observations indicate that there is other initial activation step of microglia. This might be the glutamate ionotropic receptor α-amino-3-hydroxyl-5-methyl-4-isoxazole-propionate (AMPA) type subunit 2 (GluA2)-dependent activation^52^, probably via the optic nerves in this case. An unknown initial signal may activate microglia in the OB followed by exosome secretion of sialidase. Microglia secrete sialidase from exosomes consistently. However, after activation the amounts of exosome sialidase increase^39^ followed by rapid degradation of polySia. It is noteworthy that the polySia change occurred before activation of the HPA axis and could be reversibly recovered, indicating that polySia-NCAM can be a marker for both mental disorders and for emotional changes caused by acute stress. Other than acute stress, another interesting environmental factor is the anti-SCZ drug, chlorpromazine. Chlorpromazine affects polySia expression only in the PFC, where decreased polySia expression was reported from SCZ brains^37^. The study was performed in rodents and the mechanism is still unknown. Monitoring of polySia-NCAM may provide information about the effects of drug treatments.

Acute stress has been reported to change the gamma-aminobutyric acid (GABA)ergic functions in rat brain^53^. The authors described that GABA concentration and GABA uptake were reduced in OB after 5 min of acute stress. The sensitivity of polySia to acute stress in the OB is the same and decrease in polySia expression and/or transient activity of sialidase derived from microglia may be involved in the GABAergic function in OB. In addition, an 18% decrease in the total extracellular GABA concentrations in the PFC, as determined by magnetic resonance spectroscopy (MRS), in human volunteer after acute psychological stress was described. It is considered that this is associated with a remarkably rapid pre-synaptic modulation of GABAergic input in response to acute stress^54^ and that the net effect of this reduction in GABA concentration would be to reduce the overall inhibitory influence of GABA on neural circuits involved in stress response^54^. PolySia might be involved in these phenomena, because GABA reduction in the OB and PFC are highly synchronized with polySia reduction. It is interesting that the impairments of GABAergic neurons have been repeatedly reported in stress conditions as well as in schizophrenic brains^55^. The significance of these observations require further study.

In summary, acute stress-induced decrease of polySia expression involves sialidase activity that is strongly regulated by microglial activation, especially in the OB. This change is reversible, indicating that polySia expression is highly regulated genetically and environmentally. PolySia in the sensitive areas of the brain like the OB, PFC and SCN might be useful to monitor mental conditions.

## Material and Methods

### Materials

A corticosterone enzyme-linked immunosorbent assay (ELISA) kit was purchased from Cayman Chemical (Ann Arbor, MI, USA). 2-Deoxy-2,3-dehydro-*N*-acetylneuraminic acid (DANA/Neu5Ac2en) and 4-methylumbelliferyl-α-D-*N*-acetylneuraminic acid (4-MU-Neu5Ac) were purchased from Nakalai (Kyoto, Japan). Bovine serum albumin (BSA), minocycline, α2-3,6-sialidase, anti-NCAM antibody, 0B11, astrocyte marker anti-GFAP antibody, trifluoroacetic acid (TFA), polyvinylidene difluoride (PVDF) membrane and enhanced chemiluminescence western blotting detection reagent were purchased from Merck (Darmstadt, Germany). The 12E3 polySia antibody, which recognizes the oligo/polyNeu5Ac structure (DP ≥ 5)^56^, was generously provided by Dr. Tatsunori Seki (Tokyo Medical University, Japan). ScFv735, which recognizes the polyNeu5Ac structure (DP ≥ 11)^57^, was purified as described previously^58^. Endo-*N*-acylneuraminidase (Endo-N), which cleaves the oligo/polySia structure (DP ≥ 5)^59^, was generously provided by Dr. Frederic A. Troy (University of California, Davis). Peroxidase-labeled anti-mouse Immunoglobulin G (IgG) + Immunoglobulin M (IgM) and anti-rabbit IgG were purchased from American Qualex (San Clemente, CA, USA). Anti-β-actin antibody was purchased from Santa Cruz Biotechnology (Dallas, TX, USA). CD68 activated microglia antibody and anti-NCAM antibody, ab154566 were purchased from Abcam (Cambridge, UK). Alexa488-labelled goat anti-mouse IgG and Alexa488-labelled goat anti-mouse IgM were obtained from Thermo Fisher Scientific (Waltham, MA, USA). Colominic acid, α2,8-linked polyNeu5Ac (average DP = 43), which is chemically and immunologically identical to the polySia structure in NCAM, and phenylmethylsulfonyl fluoride (PMSF) were purchased from Wako (Osaka, Japan). 1,2-Dimethylenedioxybenzen (DMB) was purchased from Dojindo Molecular Technologies, Inc. (Kumamoto, Japan). Pre-stained molecular weight marker was obtained from Bio-Rad (Hercules, CA, USA). Gabapentin (GBP)(1-(aminomethyl) cyclo-hexaneacetic acid) was purchased from Combi-Blocks (San Diego, CA, USA).

### Animals and ethics statement

Mice (C57/BL6J, male, 10 to 12 weeks of age) were obtained from Chubu Kagaku Shizai (Nagoya, Japan) and were maintained in a controlled environment (23 ± 2 °C and 50 ± 10% humidity, 12:12 light/dark cycle) with food and water available *ad libitum*. At least 1 week prior to the experiments, mice were habituated to our facilities. All procedures were approved by the Animal Care and Use Committee of Nagoya University (Permit Number: 2016022506), and performed under the relevant guidelines and regulations by the same committee. Every effort was made to minimize the number of animals used and their suffering.

### Tail suspension test

Mice were kept in the experiment room for 1 h to habituate them to the environment. After habituation, the mice were suspended over 30 cm above the floor. Mice were kept at this suspended state by their tails for 7 min. The immobility time of mice during 6 min, except for the first 1 min, was measured^60^.

### Drug treatment

DANA was dissolved at a concentration of 10 mg/ml in isotonic (0.9% NaCl) saline solution immediately before use. Mice were divided into four groups, two DANA and two control groups^61^. For the DANA groups, DANA was injected intraperitoneally (50 mg/kg)^62^. For the control groups, saline was injected. One hour after injection, one group of each DANA and control group of mice were used for the tail suspension test of acute stress as described and immediately sacrificed. The no stress groups were also sacrificed as acute stress negative groups. Minocycline (Mino) was dissolved at a concentration of 6 mg/ml in isotonic saline solution immediately before use. Mice were divided into four groups of Mino and control groups^63^. Minocycline was injected intraperitoneally (30 mg/kg). In the control group, the same volume of saline was injected. Injection was repeated once per day for one week. Two hours after final injection, mice from one treatment and control group were used for the tail suspension test and immediately sacrificed. Gabapentin (GBP) was dissolved at a concentration of 10 mg/ml in isotonic saline solution immediately before use. Mice were divided into four treatment and control groups^44^. GBP was injected intraperitoneally (100 mg/kg). In the control group, the same volume of saline was injected. One hour after injection, mice from one treatment and control group were used for the tail suspension test and immediately sacrificed.

### Sample preparation

Mice were sacrificed by CO_2_ administration immediately after the tail suspension test (Stress+ group) or after habituation in the experiment room (Stress− group). The cerebrum was surgically extracted and blood was collected. The OB was dissected and collected first. The remaining cerebrum was soaked in 1% agarose in phosphate buffered saline (PBS). After hardening, the cerebrum was coronally sliced into 500 μm sections using a Super MICROSLICER (DOSAKA EM, Kyoto, Japan) (Fig. 1c). The PFC, SCN, AMG and HIP were collected from the sections as shown in Fig. 1c. These five regions of the brain were separated and homogenized with lysis buffer (1% Triton-X100, 1 mM PMSF, protease inhibitors: 1 μg/ml aprotinin, 1 μg/ml leupeptin, 1 μg/ml pepstatin, 2 μg/ml antipain 10 μg/ml benzamidine, 1 mM ethylenediaminetetraacetic acid (EDTA), 50 mM sodium fluoride (NaF), 10 mM β-glycerophosphate, 10 mM sodium pyrophosphate and 1 mM sodium *o*-vanadate in PBS). The homogenates were incubated on ice for 1 h and centrifuged at 9,600 *g* for 15 min at 4 °C. The supernatant was collected. Protein concentrations were measured by the bicinchoninic acid (BCA) assay. For NCAM analysis, samples were de-polysialylated by incubation with Endo-N.

### Corticosterone quantification

To measure the degree of stress, blood was collected after surgery and incubated for 1 h at 25 °C and then overnight at 4 °C. The blood was centrifuged at 500 *g* for 10 min and serum was collected. The serum was diluted 1/500 in ELISA buffer. This diluted serum (5 μl) was placed in a well with 50 μl of anti-corticosterone antibody and 50 μl of corticosterone-acetylcholinesterase. The plate was incubated overnight at 4 °C. The liquid was removed and rinsed using five times using wash buffer. Reagents with acetylcholine and 5,5′-dithio-bis-(2-nitrobenzoic acid) were added (200 μl per well) and incubated for 90 min at room temperature. The absorbance was measured at 412 nm.

### Western blotting

Ten micrograms of protein from each sample were separated by 7.5% sodium dodecyl sulfate (SDS)-polyacrylamide gel electrophoresis (PAGE) and proteins were blotted on a PVDF membrane. The membrane was then blocked with PBS containing 0.05% Tween 20 (PBST) and 1% BSA at 25 °C for 1 h. The membrane was incubated with the primary anti-polySia antibody; 12E3 (2 µg/ml, mouse IgM) and 735scFv (2 µg/ml, mouse IgG), anti-activated microglia antibody, CD68 (1 μg/ml, mouse IgM) or anti-β actin antibody (2 µg/ml, mouse IgG) at 4 °C overnight. For NCAM protein analysis, anti-NCAM antibodies, 0B11 (4 µg/ml, mouse IgG) or ab154566 (1 µg/ml, rabbit IgG) were used. After washing with PBST, the membrane was incubated with secondary peroxidase-conjugated anti-mouse IgG + M antibody (1/5000 dilution) at 37 °C for 1 h. After washing with PBST, colour development and densitometry analysis were performed as previously described^25^. The exposure time depended on the antibody used. The blot used for the analysis of brain samples derived from the same brain region under the different conditions (Tail suspension (TS)−/+, Exercise (EXC)−/+, sialidase inhibitor (DANA)/Saline, minocycline (Mino)/Saline or Gabapentin (GBP)/Saline) was the same membrane. β-actin (43 kDa) was used for the control.

### Fluorometric C_7_/C_9_ analysis

To evaluate α2,8-linked oligo/polySia chains on glycoproteins blotted onto PVDF membranes, homogenates (100 μg protein as BSA) in 14 μl were added to 4 μl of 5 × Reaction buffer and 2 μl of α2-(3,6)-sialidase treatment (25 μU) and incubated at 37 °C for 1 h to release monoSia residues. The sialidase treated samples were blotted onto PVDF membranes and areas above 100 kDa were cut out. The membranes were analyzed by the fluorometric C_7_/C_9_ method for the quantification of internal sialyl residues^36^.

### Immunostaining

Mice were sacrificed by CO_2_ administration and perfused with PBS followed by 4% paraformaldehyde (PFA)/PBS. Brains were collected by surgery and fixed with 4% PFA/PBS overnight. Brains were sliced sagittally into 40 μm sections. Then sections were pretreated with 0.2% Triton-X 100 in PBS for 10 min, followed by rinsing three times with PBS and blocking with 2% BSA/PBS. Sections were then incubated with the primary antibody, anti-polySia antibody; 12E3 (5 μg/ml) and 735 (5 μg/ml) at 4 °C overnight. After rinsing with PBS, secondary Alexa 488-labelled anti-mouse antibody (1/400 dilution) was incubated at room temperature for 30 min in the dark. After rinsing three times with PBS, sections were then incubated with 4′6-diamino-2-phenyindole (1 μg/ml) at 37 °C for 10 min. After rinsing with PBS and water, sections were sealed and observed using a model BX51 confocal scanning florescent microscope (Olympus, Tokyo, Japan).

### Real-Time PCR

Total RNA was prepared from the various regions of the brain using TRIZOL® (Molecular Research Center Inc., Cincinnati, OH, USA) as previously described^64^. The first strand cDNAs were synthesized, and then quantitative real-time PCR was performed using primers (0.5 pmol each) and SYBR® GreenER™ qPCR SuperMix for iCycler premix® (Invitrogen, Carlsbad, CA, USA). PCR products were analyzed by the iCycler iQ real-time PCR analysing system (Bio-Rad). Every sample was measured in triplicate, and the gene expression levels were calculated using gene-encoding plasmids as authentic gene samples. The following specific primers were used for PCR: ST8SIA2/STX-s: ATCCTGAAGCACCATGTGAA; ST8SIA2/STX-as: ATGTGGACTTTGTTGGTCAG; ST8SIA4/PST-s: AAGGTGTAATCTAGCTCCTGTG; ST8SIA4/PST-as: TGTCATTCAGCATGGAAAGTC; NCAM-s: GCGTTGGAGAGTCCAAATTC; NCAM-as: TCATCATTCCACACCACTGAG; NEU1-s: TCAGCAATGGTACATCCTGG; NEU1-as: GTACAGAACCAACAGCTGCG; NEU2-s: ACAGAATCCCTGCTCTGCTC; NEU2-as: GTGGCTTCGTTGTAGCTTCC; NEU3-s: GAAGAACAGGACTTGGTGGC; NEU3-as: GGACCTCGTGGTCTGAAAAC; NEU4-s: ACATTCCCCATGCTTCAATC; NEU4-as: CTAGGCCATGATTCTCTGGG; Actin-s: TCCAGGCTGTGCTGTCCCT; Actin-as; TAGCCCTCGTAGATGGGCAC. For measurements, pcDNA-ST8SIA2, pcDNA-ST8SIA4, pcDNA-NCAM and pGEM-actin were used as control samples.

### Sialidase activity assay

To measure the activity of sialidase in tissues, 40 μg of tissue samples were added to the 50 μl reaction mixture including 1 ng/μl BSA and 50 μM 4MU-Neu5Ac in PBS. Solutions were incubated for 30 min at 37 °C in the dark. The reaction was stopped by the addition of 200 mM Glycine/NaOH (pH 10.4). Sialidase activity was quantified by measurement of fluorescence (excitation at 365 nm, emission at 437 nm) with the Enspire instrument (PerkinElmer, Danvers, MA, USA).

### Data analysis

All values are expressed as the mean ± SE (n is indicated) and p-values were evaluated by the student t-test. Effect size was evaluated by Cohen’s d.

## Supplementary information

SI

## Data Availability

All data generated or analysed during this study are included in this published article and its Supplementary Information files.
