# Supplementary material for: Acute stress-induced change in polysialic acid levels mediated by sialidase in mouse brain
Source: Sci Rep. 2019 Jul 9;9:9950. doi: 10.1038/s41598-019-46240-6 (PMC6616613; doi:10.1038/s41598-019-46240-6)

Acute stress-induced change in polysialic acid levels mediated by sialidase in mouse brain

Chikara Abe, Yang Yi, Masaya Hane, Ken Kitajima, Chihiro Sato*

Bioscience and Biotechnology Center, Nagoya University, Chikusa, Nagoya 464-8601, JAPAN.
*Correspondence to: C. S. (chi@agr.nagoya-u.ac.jp)

Legend for Supplementary Figures

Supplementary Figure 1. PolySia expression in specific brain regions. (a) OB, PFC and SCN derived from acute stress treated (TS+) or non-treated (TS-) mice were immunostained with anti-polySia (12E3 for SCN) and 735 for (OB and PFC) antibodies. (b) Whole gel blots of brain samples derived from the five brain regions (OB, PFC, SCN, AMG and HIP). Portions of the immunostained gel pattern are shown in Fig. 2b. TS indicates tail suspension. M1~5 indicate the animal number used in each condition. X is the lane that was used for other experiments for reduction of animal numbers. PEB is a homogenate that was derived from a pig embryonic brain. PEB is the positive control for anti-polySia staining. The β-actin band (43kDa) was used for the loading control. The polySia-NCAM smear band (greater than 150kDa area, see Fig. 2b) was used for the evaluation. (c) Blots of anti-NCAM antibody and anti-β actin antibody using endo-N treated brain samples derived from five brain regions (OB, PFC, SCN, AMG and HIP). TS indicates the tail suspension. M1~5 indicate the animal number used in each condition. X is the lane that was used for other experiments for reduction of animal numbers. The β-actin band (43kDa) was used for the loading control. The NCAM bands (140kDa and 180 kDa) were used for the evaluation in Fig. 2e.

Supplementary Figure 2. Effect of exercise on polySia expression in mice brains. Immunoblot of polySia staining using anti-polySia antibody used in Fig. 3c. Homogenates of OB and PFC derived from mice before (EXC-) and after exercise (EXC+) were analyzed by western blotting using anti-polySia antibody and anti-β-actin antibody (n=3/each group). For each brain area, the blot was obtained by the same SDS-PAGE/immunoblotting. The blot was further cut into the upper and lower parts for anti-polySia and anti-actin antibodies, respectively. The β-actin band (43kDa) was used for the loading control. The polySia-NCAM smear band (greater than 150kDa area) was used for the evaluation. The blots corresponding to these blots for each brain area were shown in Fig. 3c.

Supplementary Figure 3. Effects of sialidase inhibitor on acute stress-induced polySia expression. (a) Sialidase activity. Measurement of sialidase activity using mice pretreated with saline or DANA before and after acute stress (TS- and TS+) (n=5). Sialidase activity using homogenate of olfactory bulb and prefrontal cortex were analyzed using 4MU-Neu5Ac. Saline/TS- group was set to 1.0. (t-test) (n=5). (b) Immunoblots of polySia staining. Mice were pretreated with saline or DANA. Half of the mice were exposed to acute stress (TS+) and the other half were not exposed (TS-). PolySia expression in OB and PFC was evaluated by western blotting using anti-polySia antibody used in Fig. 6c. For each brain area, the blot was obtained by the same SDS-PAGE/immunoblotting. Each blot was further cut into the upper and lower parts for anti-polySia and anti-actin antibodies, respectively. M1~5 indicate the animal number used in each condition. The β-actin band (43kDa) was used for the loading control. The polySia-NCAM smear band (greater than 150kDa area) was used for the evaluation. PEB is a homogenate that was derived from a pig embryonic brain. PEB is the positive control for anti-polySia staining.

Supplementary Figure 4. Real-time PCR of sialidase genes before and after acute stress. The amounts of sialidase genes (Neu1~Neu4) in OB and PFC were evaluated by real-time PCR. Gene expressions of TS- (without acute stress) was set to 1.0. (n=3, t-test).

Supplementary Figure 5. Effects of microglia inhibitor on acute stress-induced polySia expression. Mice were pretreated with saline or Mino. Then half of the mice were exposed to acute stress (TS+) and the other half were not exposed (TS-). Activated microglia were analyzed by western blotting using anti-CD68 antibody used in Fig. 7c (a). For each brain area, the blot was obtained by the same SDS-PAGE/immunoblotting. Each blot was further cut into the upper and lower parts for anti-CD68 antibody and anti-actin antibodies, respectively. M1~5 indicate the animal number used in each condition. The β-actin band (43kDa) was used for the loading control. The CD68 band (60kDa) was used for the evaluation. (b) PolySia expression in OB and PFC were evaluated by western blotting using anti-polySia antibody used in Fig. 7d. For each brain area, the blot was obtained by the same SDS-PAGE/immunoblotting. Each blot was further cut into the upper and lower parts for anti-polySia antibody and anti-actin antibodies, respectively. M1~5 indicate the animal number used in each condition. The β-actin band (43kDa) was used for the loading control. The polySia-NCAM smear band (greater than 150kDa area) was used for the evaluation. PEB is a homogenate that was derived from a pig embryonic brain. PEB is the positive control for anti-polySia staining.

Supplementary Figure 6. Effects of astrocyte inhibitor injection on polySia expression during acute stress. (a) Astrocytes were analyzed by western blotting using anti-GFAP antibody. The blot was obtained by the same SDS-PAGE/immunoblotting. The blot was immunostained with anti-GFAP. After deprobing, the membrane was re-immunostained with anti-actin antibody. The β-actin band (43kDa) was used for the loading control. The GFAP band (50kDa) was used for the evaluation. (b) PolySia expression in PFC were evaluated by western blotting using anti-polySia antibody. For PFC, the blot was obtained by the same SDS-PAGE/immunoblotting. Each blot was further cut into the upper and lower parts for anti-polySia antibody and anti-actin antibodies, respectively. M1~5 indicate the animal number used in each condition. The β-actin band (43kDa) was used for the loading control. The polySia-NCAM smear band (greater than 150kDa area) was used for the evaluation. PEB is a homogenate that was derived from a pig embryonic brain. PEB is the positive control for anti-polySia staining.

Supplementary Figure 1 (Abe et al.)

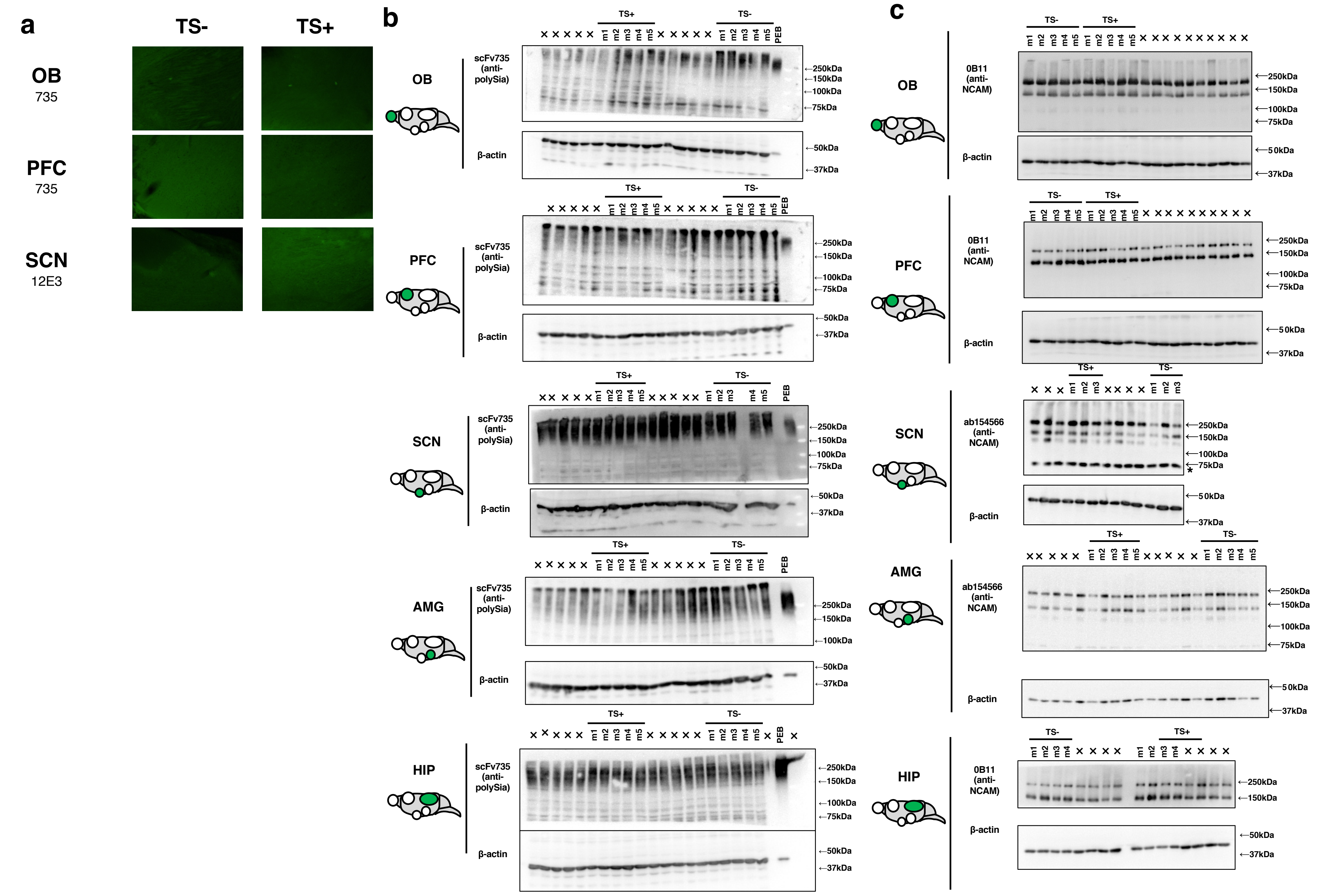

Supplementary Figure 2

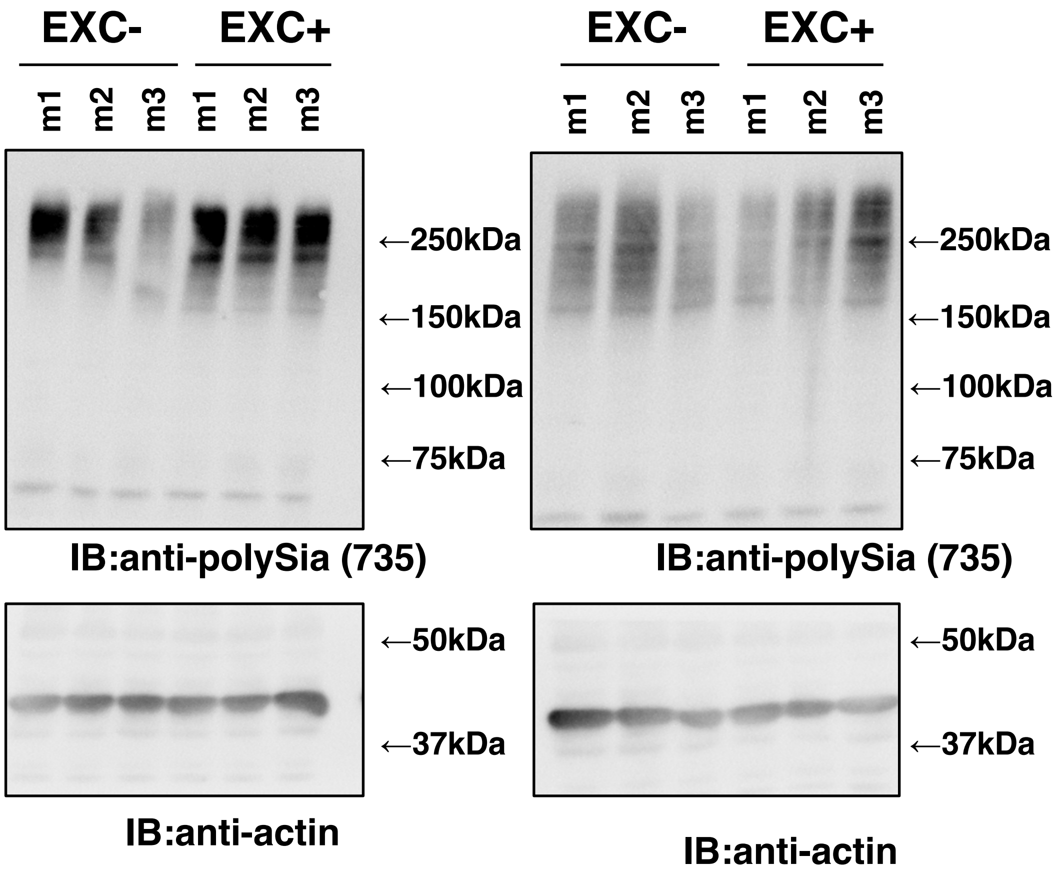

Supplementary Figure 3

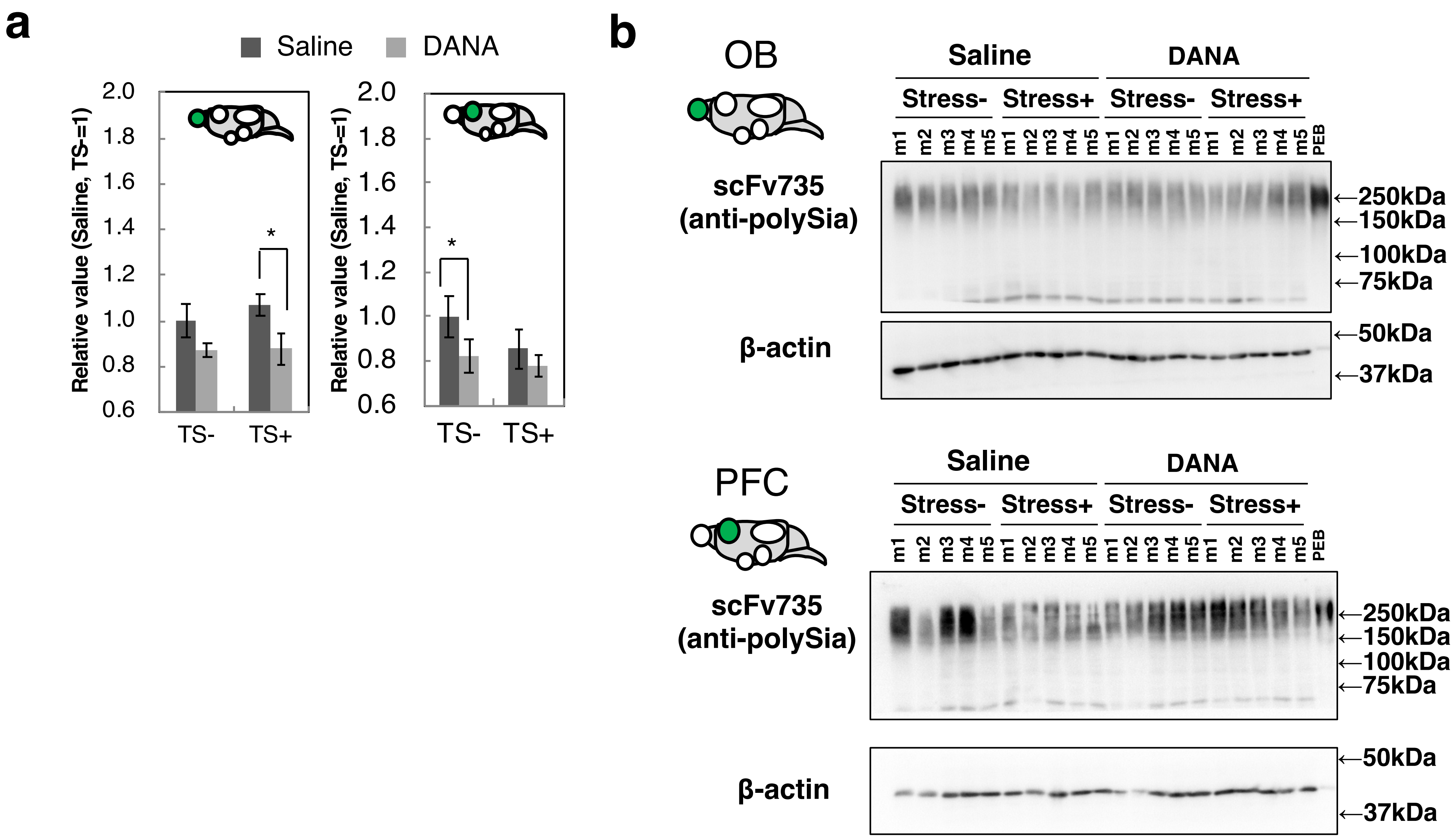

Supplementary Figure 4

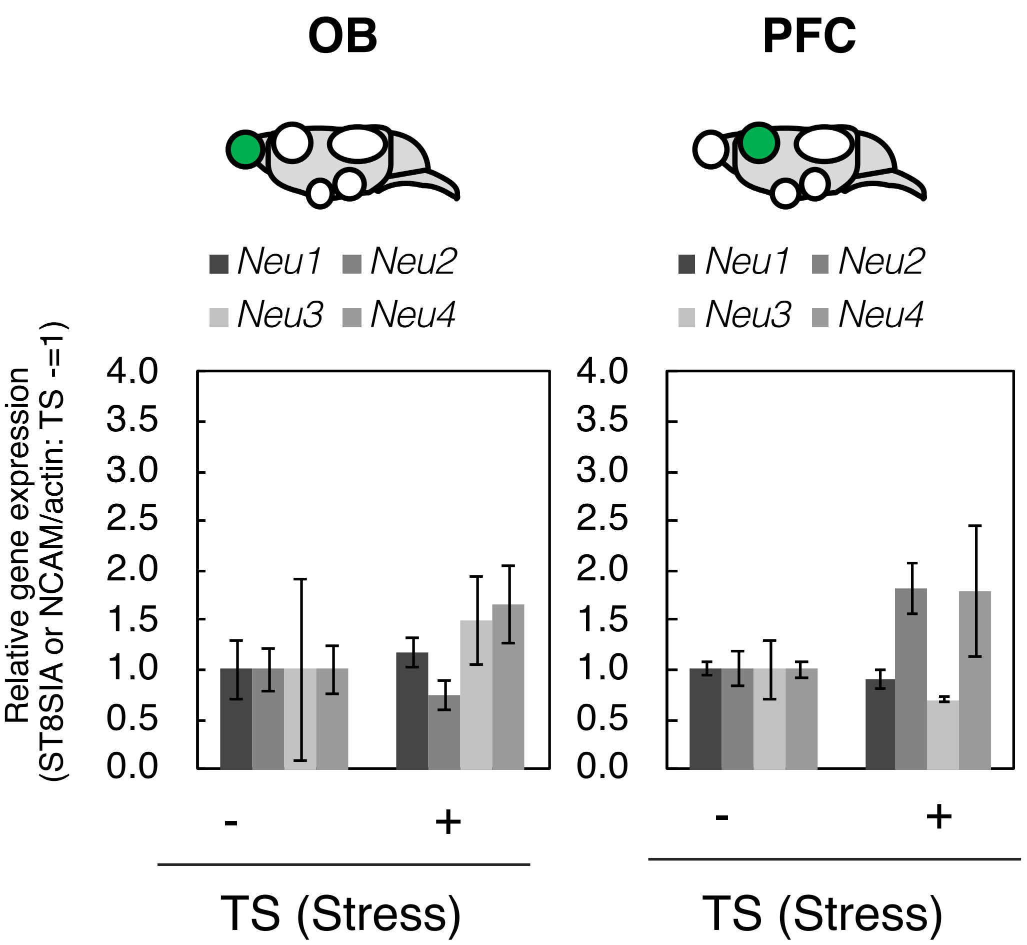

Supplementary Figure 5

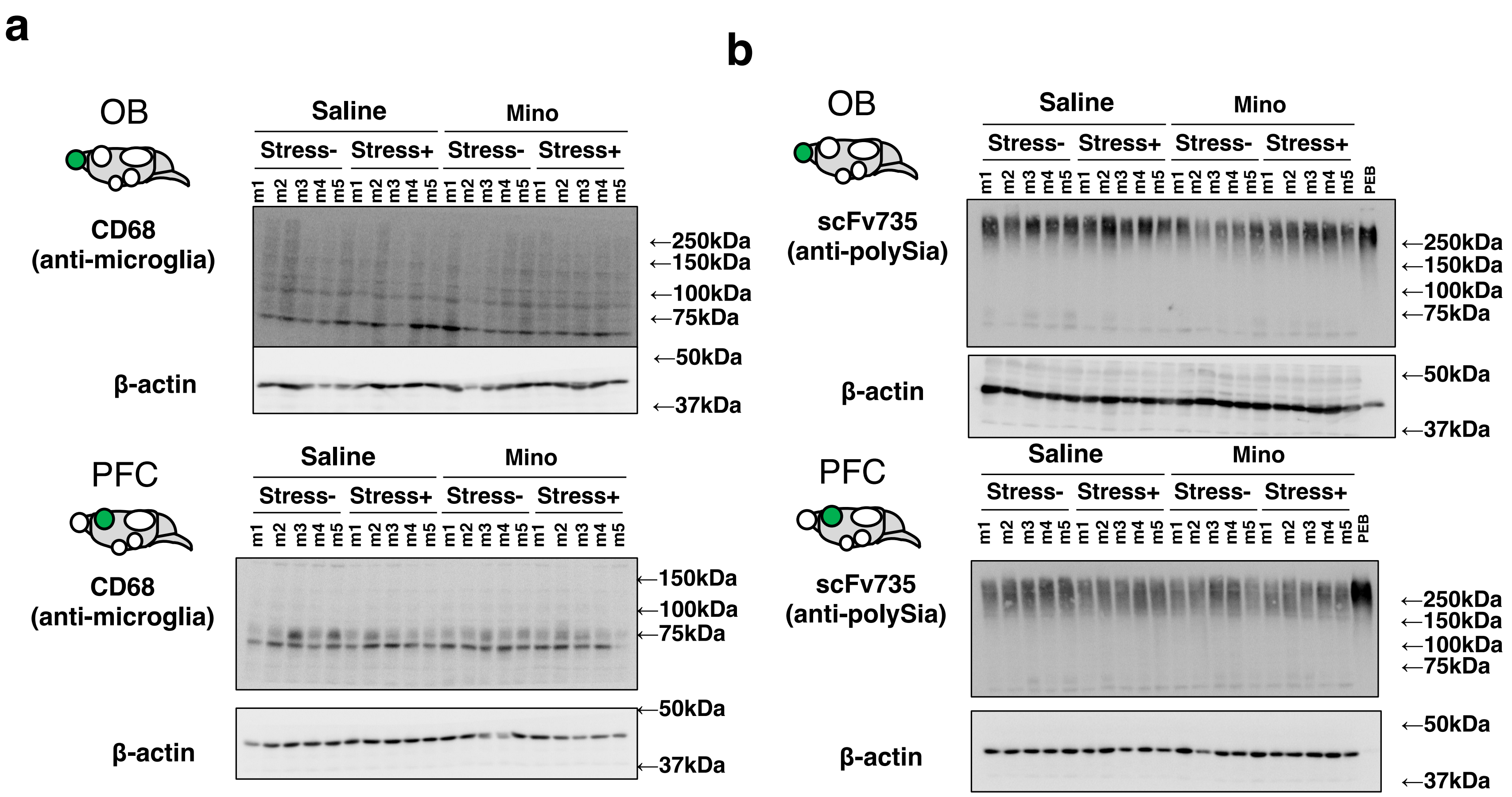

Supplementary Figure 6

a

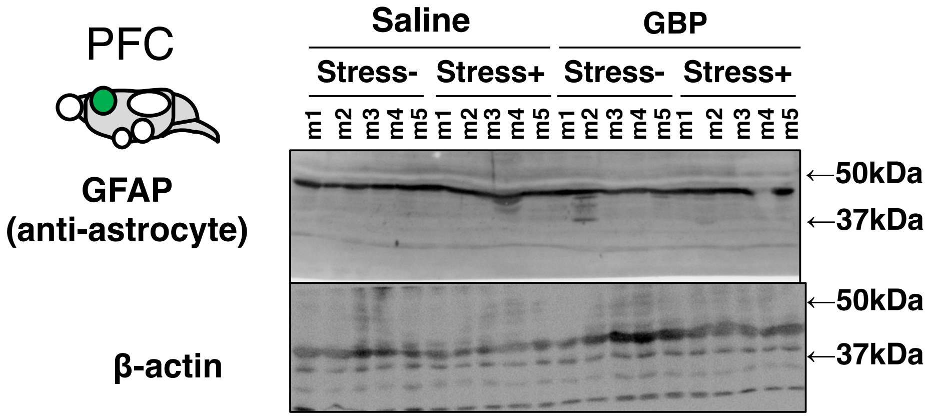

b

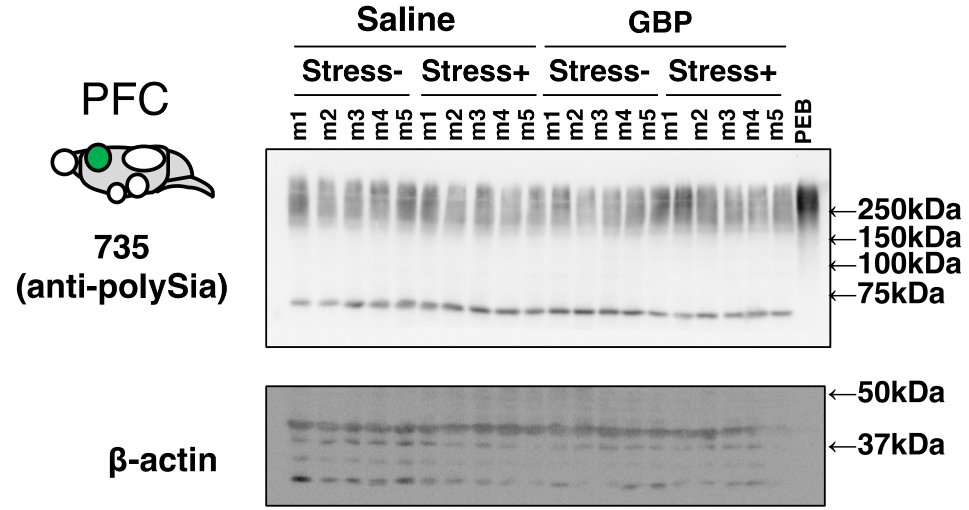

Supplement: Supplementary file 1 — SI [file 41598_2019_46240_MOESM1_ESM.pdf]
